# Structural DNA Nanotechnology: From Design to Applications

**DOI:** 10.3390/ijms13067149

**Published:** 2012-06-11

**Authors:** Reza M. Zadegan, Michael L. Norton

**Affiliations:** 1 Center for DNA Nanotechnology (CDNA), Interdisciplinary Nanoscience Center (iNANO), Aarhus University, 8000 Aarhus C, Denmark; 2 Department of Chemistry, Marshall University, Huntington, WV 25755, USA

**Keywords:** DNA nanotechnology, self-assembly, nanostructures, DNA origami

## Abstract

The exploitation of DNA for the production of nanoscale architectures presents a young yet paradigm breaking approach, which addresses many of the barriers to the self-assembly of small molecules into highly-ordered nanostructures via construct addressability. There are two major methods to construct DNA nanostructures, and in the current review we will discuss the principles and some examples of applications of both the tile-based and DNA origami methods. The tile-based approach is an older method that provides a good tool to construct small and simple structures, usually with multiply repeated domains. In contrast, the origami method, at this time, would appear to be more appropriate for the construction of bigger, more sophisticated and exactly defined structures.

## 1. DNA Self-Assembly

DNA (deoxyribonucleic acid) is the molecule of choice for designed nanostructure fabrication since it has excellent features such as molecular recognition, self-assembly, programmability, predictable nanoscale structure and is easily synthesized [1]. DNA self-assembly occurs through the specific base-pairing of complementary bases. Unique geometries can be formed when complementary single-stranded DNA molecules anneal together to form double-stranded DNA molecules by design. This complementary binding only happens if the strands are complementary over a sufficient number of bases, the temperature is appropriate and the required ions are present at the optimum concentration.

**Figure 1:**
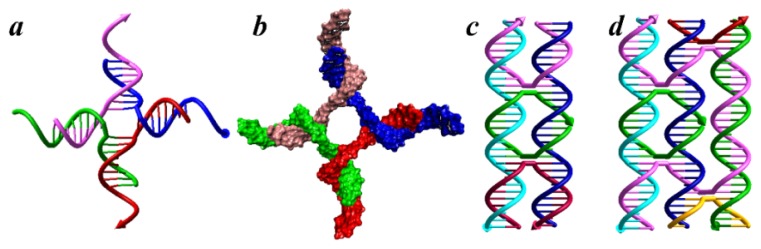
Basic DNA structures for tile-based self-assembly. (**a**) Four-arm junction with sticky ends; and (**b**) Its detailed molecular model; (**c**) Double crossover motif (DX); (**d**) Triple crossover motifs (TX).

Fabrication of DNA nanostructures was first proposed by Nadrian C. Seeman in the 1980s [2]. Inspired by the Holliday Junction, which occurs as a recombination intermediate in the cell [3], he proposed a number of DNA nanostructures using DNA branched junctions (Figure 1a,b). He optimized the sequences to make the structures thermodynamically stable [2,4]. Later he designed and generated much more complex DNA structures with different arrangements (parallel isomers) of the double crossover motif (DX) (Figure 1c), triple crossover motifs (TX) (Figure 1d), two-dimensional lattices, and polyhedral DNA structures such as the cube, six-connected network, pentagonal dodecahedron and truncated octahedron [5,6].

## 2. Tile-based DNA Structures and Applications

By applying the tile-based motifs and optimizing them, Winfree *et al.* [7], LaBean *et al.* [8] and Feng *et al.* [9] introduced DNA lattices. Other studies have been conducted to optimize and use the tile based DNA assembly for different applications [10]. Construction of large one dimensional (1D) and two dimensional (2D) DNA arrays have been demonstrated by using the DNA tiles with sticky ends [11–13]. Formation of other structures such as 4 by 4 cross tile [14], tensegrity triangle [15] and six-helix tube [16] have been reported. Liu *et al.* reported the formation of 2D DNA crystals by using only two DNA strands [17], and He *et al.* reported construction of DNA nano-arrays by using three oligos and implementing sequence symmetry [17]. A report by Zheng *et al.* demonstrated formation of three dimensional (3D) DNA crystals using tensegrity triangles as the building blocks [18].

Novel motifs are continuously being introduced, and recently, a half-crossover based structure was reported by Yin *et al.* [19], which allowed them to form DNA tubes composed of four-helix bundles all the way up to twenty-helix bundles. In another work, Hansen *et al.* introduced a way to incorporate some features of both the origami method and the tile based self-assembly to produce weave tile structures [20]. They reported formation of flexible structures that were used to increase the anticoagulant activity of thrombin-binding aptamers. The structure was composed of two long strands that have complementarities for each other (woven strands) and possess the sticky ends that provide complementary sequences for other tiles to produce the lattices. Unlike the other tile based or origami methods, the weave tile structure does not form based on the holiday junctions or crossovers and just benefits from the exact design of the structure based on the geometry of the DNA bases. The new design solves the problem of incorrect strand stoichiometry that has been reported with other tile-based assemblies because this approach only uses two DNA strands.

The DNA tile based system has been used to make 3D structures. The first tile based 3D DNA structure was introduced by Seeman [21]. However, the study only provided indirect evidence for the formation of the 3D structure. A few years later other studies reported the construction of 3D DNA structures. An interesting study by Shih *et al.* reported formation of an octahedron by self-folding of a 1.7 kb single-stranded DNA (heavy strand) and a few smaller strands (light strands) [22]. This structure might be considered as an early example of the DNA origami technique. Another study by Goodman *et al.* reported construction of a tetrahedron DNA structure. They have also reported conformational changes in the structure due to restriction enzyme digestions [23] or by strand displacement [24]. Formation of various 3D structures such as triangular, cubic, pentameric and hexameric prisms was reported by Aldaye and Sleiman [25]. Another interesting study by He *et al.* reported the formation of tetrahedron, dodecahedron, buckyball structures [24] and a DNA octahedral structure [26].

The addition of dynamic properties to DNA nanostructures is another practice which has been applied over the last decade [23,24,27–32]. One of the most often employed strategies to trigger and produce dynamics in DNA nanostructures is strand displacement, which is a process of displacing pre-hybridized strands (one or more) in a DNA complex (consisting of two or more strands) via partial or full hybridization with a new strand (usually called a displacement strand), with a longer region of complementarity. To start strand displacement, the displacement strand hybridizes a single-stranded complementary region in the DNA complex and by branch migration displaces the pre-hybridized strand. The single-stranded region in the initial complex is called a toehold.

Toehold based strand displacement in DNA nanotechnology was initially proposed by Yurke *et al.* [32]. They demonstrated a nanodevice which switched from open to closed states and *vice versa* by multi step hybridization and strand displacement. Later, scientists have programmed the movement of DNA molecules based on toehold strand displacement. Shih and Pierce demonstrated walking of a DNA molecule on top of a DNA tube driven by fuel strands [29], which has been followed by more DNA walker studies [33–36]. In another work a series of ligation and cut processes were used to force the DNA walker to move along a longer strand of DNA [31]. Recently, Wang *et al.* introduced a new strategy for stepwise walking of a bipedal walker, in which they triggered the forward step by introducing Hg^2+^ and H^+^ ions and the backward step by introducing OH^−^ ions and cysteine [30].

Tile-based DNA nanostructure architecture is a promising method, but there are major drawbacks for the tile-based assembly strategy. First, the design of complex structures using the tile method is a challenge since one needs to design and check the new sequence for each step, which is a time consuming and problematic step. Secondly it is very hard to control production of complex high order nanostructures, and even though some of the structures have finite size and shape, many other structures such as arrays or grids grow as long as sticky strands are available, and therefore there is no control over size. Finally, in order to obtain the predicted structure the strands need to be highly quantitatively controlled.

## 3. Origins of DNA Origami

The “DNA origami” method was first proposed and implemented by Paul W. K. Rothemund in 2006 [37], in which he folded a long viral single-stranded DNA (ssDNA) molecule to create DNA structures of arbitrary shapes. However, DNA origami was foreshadowed by at least two other prior works. In the first attempt, Yan *et al.* reported formation of the nano arrays by using a long scaffold and some shorter strands [38]. However, they could not demonstrate exact control over size and shape of the structures. Another advance, mentioned previously, was published by Shih *et al.* who reported formation of an octahedron by self-folding of a long single-stranded DNA and a few smaller strands [22]. Moreover, a long time ago Williamson suggested the term “RNA origami” for 3D structures that could be formed by self-folding of the RNA molecules [39].

**Figure 2:**
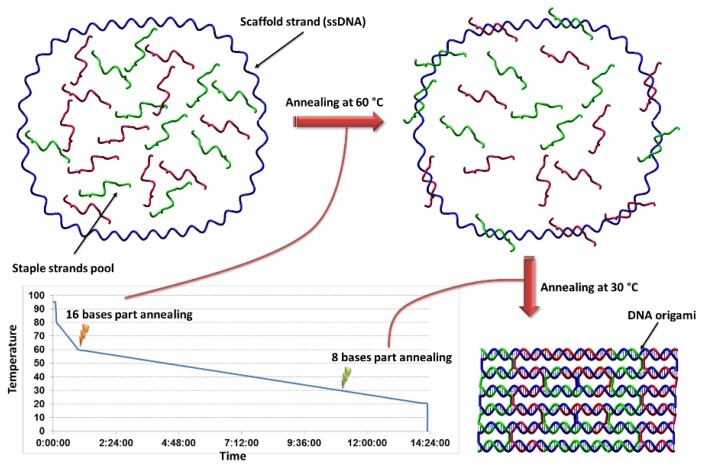
Schematic illustration of DNA folding in origami construction. A long ssDNA (usually circular) is treated with an excess number of shorter complementary strands, so called “staple strands”. Sample annealing chart in the DNA origami construction is shown in the lower-left. The annealing process starts with heating of the solution, followed by fast cooling to 80 °C with further cooling to 60 °C in a slower process. The sample is cooled down very slowly to 20 °C.

The term origami refers to the Japanese folk art of folding paper into a special shape. The method is called DNA origami since one long strand of DNA is folded to produce the desired structure by the help of smaller staple strands. The origami folding process is described in Figure 2; the method is based on folding of the large ssDNA (usually the 7.3 kilobase genome of the M13 bacteriophage) with an excess of smaller complementary strands (typically 32 bases). These small strands are called “staple” strands and are complementary to at least two distinct segments of the long ssDNA. Long ssDNA and an excess of staple strands are then heat-annealed in a specific buffer with high concentration of magnesium to form the origami.

## 4. 2D DNA Origami

The origami method is applied to construct 2D and recently 3D DNA nanostructures. In the revolutionary and earliest work [37], Rothemund illustrated construction of many arbitrary structures such as stars, squares, rectangles, smiley faces, triangles and some other complex structures. He was also able to demonstrate the addressability of the structures by showing formation of designed patterns on the top of the origamies. Only a few months after Rothemund’s original origami work was published, a Chinese group reported construction of a map of china by this method [40]. In 2008 software to design arbitrary structures, called SARSE, was released [41], and it was rapidly followed by caDNAno, an improved tool for the design of 2D and 3D DNA origami [42]. More recently, canDo [43], an online program to predict properties, such as flexibility and predicted final shape of designed structures, was developed by the Dietz group.

Formation of DNA origami from a double stranded DNA (dsDNA) scaffold has been reported [44] which opened a new door in the DNA origami method, the use of a broader range of scaffolds. Li *et al.* reported a tile assembly of the origamies to make higher order self-assembled DNA nanostructures [45]. Very recently Endo *et al.* reported formation of multi-domain DNA origami by using origami four-way junctions [46], and in a very similar work Rajendran *et al.* reported construction of the multi-domain DNA origami [47]. The DNA origami method is successful and popular since it does not require any sequence design, hard time-consuming stoichiometry studies or control over the quality and quantity of the staple strands, which are main challenges in tile based DNA architecture.

DNA origamies are interesting since they can be used as platforms for the study of other systems. In the earliest work, patterns (hairpin dumbbells) on top of the origamies were imaged by AFM [37]. DNA origami was used as a template for patterning using streptavidin molecules [48] and Shen *et al.* showed patterning of enhanced green fluorescent protein on top of the origami [49]. Kuzyk *et al.* demonstrated patterning of the coat of arms of Ukraine by putting streptavidin molecules on top of a rectangular origami [50]. Protein decoration of DNA origami has also been reported by Sacca *et al.* [51]. Additionally, virus capsids have been immobilized on top of DNA origamies [52].

DNA origami has been used to investigate binding of thrombin molecules to their aptamers [53]. Moreover, patterning of gold nanoparticles has been reported on the six-helix bundle DNA origami [54] and rectangular origami [55]. By placing fluorophores at specific positions of DNA origami as a ruler, calibration objects for super-resolution optical microscopy have been reported [56]. DNA nanotube origamies have been aligned between gold islands [57] and recently, Liu *et al.* reported gold metallization of branched DNA origami [58].

In a very interesting study, Manue *et al.* [59] showed positioning of single-walled carbon nanotubes on top of rectangular origami. They demonstrated stable field-effect transistor-like behavior of the structure, which is an important advance toward using a complex system of DNA and nanotubes in nanoelectronics. Eskelinen *et al.* positioned carbon nanotubes on top of the DNA origami by aid of interactions between streptavidin molecules and biotinylated DNA strands precisely positioned on the origami and wrapped around the carbon nanotube [60]. In another major step toward wafer scale origami applications, DNA origamies have been placed and oriented on lithographically patterned surfaces thus combining the top-down and bottom-up approaches [61]. Furthermore, Hung *et al.* reported specific positioning of gold nanoparticles on top of lithographically confined DNA origamies [62]. Positioning and alignment of origami between nanoelectrodes has been demonstrated using dielectrophoretic trapping [63]. Kuzyk *et al.* attached gold nanoparticles to a tube shaped origami and showed formation of plasmonic nanostructures. The exact positioning capability intrinsic to the DNA origami method enabled them to incorporate nanoparticles at very precise positions and thus they could study and prove predicted optical properties of the nanostructures [64]. Ke *et al.* demonstrated label free detection of RNA molecules by an AFM study of hybridization of the target on top of a rectangular origami [63]. In a very similar study the hybridization of DNA target to the probe on the surface of the origami was monitored using AFM microscopy [63]. In very recent work, detection of the single nucleotide polymorphisms has been reported using DNA origami [65]. DNA origami also has been used to seed algorithmic self-assembly, by assembling DNA tiles on a seed of special sticky ends at the edge of a 2D DNA origami [66,67]. DNA origami has been used to study the effect of double-stranded DNA tensions on the DNA methyltransferase efficiency [68] and by using DNA origami, Subramani *et al.* reported a new secondary DNA binding site in the enzyme topoisomerase I [69].

In a very interesting development, scientists from Hao Yan’s and Erik Winfree’s groups demonstrated a molecular robot which moves along a predefined path on top of a rectangular origami [70]. The movement of this walker is based on a simple enzymatic reaction. In another advance, Gu and colleagues from Seeman’s group introduced a DNA walker molecule which moves on top of an origami in a programmed path and collects cargos which are placed at specific positions on the origami [71]. In another study Wickham *et al*. programmed directed, uniform and continuous translation of a molecular motor along a 100 nm track on flat DNA origami [72].

## 5. 3D DNA Origami

A number of studies reported the production of 3D DNA origami structures in 2009. William Shih is a pioneer in this field and one might consider the production of an octahedron as the first successful attempt to make 3D DNA origamies [22]. However, at the time the term “DNA origami” still was not established.

**Figure 3:**
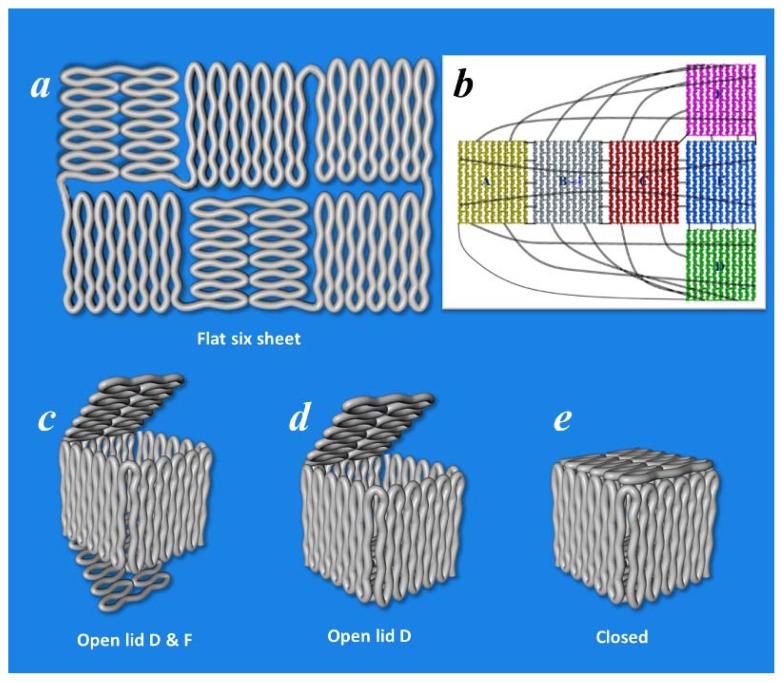
3D DNA box origami. (**a**) Six flat square-shaped origami domains which by their connection (black lines in (**b**)) will form a 3D DNA box origami; (**c–e**) DNA box origami in different states.

There are several strategies for assembling 3D origamies. One method is based on folding flat surfaces against one another through stacking of the helices in separate domains of a flat multi domain origami. The links between the helices could be coaxial, noncoaxial, orthogonal or at any angle. This strategy has also been used to structure complex 2D origami, for example to produce a triangle [37] and three, four and six flat origami faces [73]. This strategy has also been used to make 3D structures such as three, four and six sided prisms [73], icosahedrons [74], 3D DNA box with an openable lid [75] (Figure 3), 3D DNA box origami [76] and a tetrahedron [77].

**Figure 4:**
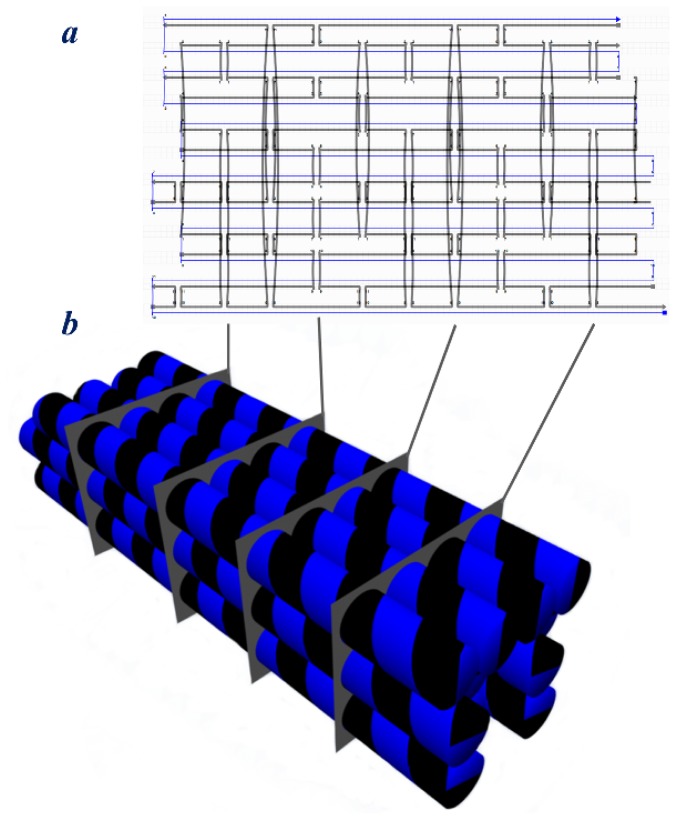
3D solid DNA origami structures; (**a**) flat design of the crossover pattern for the designed structure. Blue line represents the scaffold and the black lines represent the staple strands; (**b**) Schematic model of the multilayer 3D origami design where three layers of DNA helices are connected via many crossovers. The crossover patterns for each segment are illustrated in (**a**).

Another strategy is to make 3D structures out of multiple layers of DNA origami (Figure 4). Shih and his coworkers reported formation of various solid 3D DNA origamies, such as solid multilayer squares [75], square nut, monolith, stacked cross, slotted cross, railed bridge and a genie bottle [42,74]. They even demonstrated formation, by design, of bent and curved solid structures including gear shaped, spiral concentric multilayer tubes, beach ball-like and other 3D DNA origamies [78]. These designs are based on crossovers between neighbor helices on either honeycomb lattices [74] or square lattices [77]. The honeycomb or hexagonal based design is more useful when greater rigidity of the structure is desired, and the square based design leads to the formation of dense structures with flatter surfaces. Although the multilayer origami methods provide more rigid and stable structures, multilayer origami require longer than usual annealing times to assemble, most likely due to hindered diffusion inside the growing structures.

Shih’s group reported design and formation of twisted structures, resulting from the insertion or deletion of bases in between the crossovers [78]. A year later a novel concept was introduced by Han *et al.* who showed reconfiguration of twisted DNA nanostructures [79]. They created Möbius DNA strips that by displacement of specific strands could reconfigure to another structure. The authors call this technique “kirigami” after the Japanese art of paper cutting. In another work, Li *et al.* reported production of a 3D tetrahedral DNA structure which formed from one DNA strand [78]. This might be considered a real single molecule DNA origami since no elements other than the scaffold strand participates in the folding of the structure. They also have successfully replicated the structure *in vivo*, which is a masterful achievement in developing the origami method, since it raises the hope of large scale production of high quality origamies at relatively low cost.

## 6. Discussion

DNA is a unique molecule for generating nanoscale molecular architectures, with a large number of useful features that facilitate molecular engineering. The tile based method is a promising method when dealing with small and non-complex structures or structures with repeating building blocks. Although tile based structures provided us with a large number of useful and interesting learning steps, the drawbacks of the method necessitated the investigation of another DNA structural method, called DNA origami. The origami method provides a free hand method to design and to construct more sophisticated and highly addressable structures. Currently, using the DNA origami method, construction of large, highly-ordered and complex structures consisting of several hundred thousand atoms (usually about 5 megaDaltons) is not only possible, but relatively easily accomplished. The DNA origami method is a very useful tool to generate precisely defined molecular systems for characterization. Some of the demonstrated applications of DNA origami include molecular pattering of surfaces, directed positioning of particles/materials, construction of nanorobots and carriers, drug and material delivery, molecular recognition and sensor applications.

An example of a high impact application of DNA origami, an enclosed, “smart” molecular nano-carrier, was recently reported by Douglas *et al*. [80]. They produced a reconfigurable DNA origami structure which exploited the lock-key system, inspired by the previously described reconfigurable DNA box origami [75], to open the locked structure. They proved that after addition of the right keys the loaded cargo material was exposed to the medium. Such a nanorobot could be used in DNA computation and nanoelectronics in addition to this most interesting application in combined cell-targeting and drug delivery. In another report, a mechanical machine to detect target molecules was described by Kuzuya *et al*. [81], in which binding of the target molecule to the DNA origami changes the configuration of the DNA origami structure and this reconfiguration was successfully detected by fluorescence techniques and via AFM imaging. This work provides another high impact example of the applications of DNA origami.

However the tile-based and origami methods still suffer from major drawbacks. DNA origamies are not stable in various conditions and require special care. For example, pH has a drastic effect on the structure of DNA nanostructures. In low pH solutions the DNA may be de-purinated and in high pH the hydrogen bonding between the DNA strands will be disrupted. Heating, many chemicals and some organic solvents denature double-stranded DNA. DNase enzymes destroy DNA strands by catalyzing the cleavage of the DNA backbone. Thus handling and storing of samples consisting of DNA structures must be performed carefully. The ions present in solution have a strong impact on DNA structures; at low ionic strength the DNA structures will decompose, and high salt concentrations lead to aggregation of the structures. Molecular tensions and mechanical forces may have negative effects on structures, particularly on extended structures, too.

Today, production of DNA origami is based on the use of a very limited number of different scaffolds. To make more sophisticated structures, developing new scaffold production methods will be necessary. A few years ago, Högberg *et al.* proposed production of origamies from double-stranded DNA, a method that may aid in the use of various scaffold sources [44]. Moreover, to make more complex DNA structures, more highly developed computer programs are necessary. Currently, only a limited number of software programs have been developed to assist with designing complex DNA structures [41,42,82,83], and to predict the structural properties of the constructs [43,83,84]. Each of the current programs has its advantages and disadvantages. Additionally, sequence optimization is very important and should be performed in concert with structure design. In conclusion, DNA nanotechnology and recently DNA origami have opened a novel pathway for addressing many previously impossible challenges.
